# Predictors of Source Memory Success and Failure in Older Adults

**DOI:** 10.3389/fnagi.2019.00017

**Published:** 2019-02-05

**Authors:** Selene Cansino, Frine Torres-Trejo, Cinthya Estrada-Manilla, Liuba Ramírez-Barajas, Miguel Pérez-Loyda, Aidé Nava-Chaparro, Mariana Hernández-Ladrón-deGuevara, Silvia Ruiz-Velasco

**Affiliations:** ^1^Laboratory of NeuroCognition, Faculty of Psychology, National Autonomous University of Mexico, Mexico City, Mexico; ^2^Applied Mathematics and Systems Research Institute, National Autonomous University of Mexico, Mexico City, Mexico

**Keywords:** episodic memory, source memory, recollection, aging, logistic regression

## Abstract

Source memory decline has been identified as one of the types of memory most seriously affected during older age. It refers to our capacity to recollect the contextual information in which our experiences take place. Although most elderly adults will be affected by progressive source memory decline, a subset of individuals will not follow this average pattern; instead, their source memory capabilities will remain functional. Likewise, a minority of individuals will manifest an extreme decay of their source memory abilities. The objective of the present study was to identify among 120 potential predictors that significantly contributed to these two extreme source memory outcomes. Spatial source memory was measured in a sample of 519 healthy individuals between 61 and 80 years old. Individuals who performed below the 20th and above the 80th percentiles in the source memory task were defined as individuals whose episodic memory failed and succeeded, respectively. Logistic models identified five and six significant predictors of source memory success and failure in older age, respectively. High source memory performance was mainly predicted by healthy cardiovascular markers and psychological traits, whereas low source memory performance was primarily predicted by consumption habits and by less engagement in mental activities. The models identified relevant biological and life experiences that underlie these unusual source memory outcomes in older age.

## Introduction

The fact that several cognitive functions deteriorate with advancing age has prompted interest in investigating possible actions to mitigate such decline. Within this main purpose, the examination of older individuals with preserved cognitive functions is of particular interest to search for predictors of the retention of cognitive status. However, the advancement of this research field has encountered several problems. One problem is that only a few persons experience this condition; therefore, the examination of large samples is often needed to encounter individuals with this characteristic. As an example, one study (Habib et al., 2007) found that only 3% from a sample of 1463 participants were classified as high-functioning at the age of 70 years old or older.

Another difficulty arises from the different criteria used to identify older adults with preserved cognitive abilities. Rowe and Kahn (1987, 1997) suggested the term successful aging to encompass individuals with a low possibility of suffering diseases or disabilities related to diseases, high cognitive and physical performance, and actively engaged in life activities. However, this definition refers to the aging process in general and is based on ill-defined criteria. Conversely, more precise benchmarks have also been outlined; for some authors (Habib et al., 2007), high functioning older adults are those who perform in cognitive tasks from several domains above the mean performance of middle-aged adults (50–65 years old) and maintain this performance over 5 years. Others (Cabeza et al., 2002) demand older adults to perform similar to young adults in several tasks to be considered high-performing older adults. Similarly, adults that perform in the upper one-third of the entire sample of older adults examined using different tests have been classified as successfully aging individuals (Inouye et al., 1993; Albert et al., 1995; von Faber et al., 2001; Andrews et al., 2002). A requirement of cognitive stability over time has also been proposed as a criterion to identify successful aging (Liang et al., 2003). Notably, none of these criteria are based exclusively on one cognitive domain. This approach provides useful information about general cognitive functioning, even though cognitive decline is not equivalent across processes and is even different within the same domain.

The finding that performance variability increases in several cognitive tasks with advancing age (Hultsch et al., 1998; Christensen et al., 1999; Wilson et al., 2002) provides evidence that some individuals experienced a sharp decline, while others maintained their cognitive abilities. Previous studies have assessed individuals who have successfully preserved their cognitive functions (e.g., Albert et al., 1995; Habib et al., 2007), but those who have suffered from a severe cognitive decay, while still remaining in the range of normal functioning, have been less examined in the past (Berkman et al., 1993; Ylikoski et al., 1999).

Some of the predictors of successful aging that have been identified are education (Berkman et al., 1993; Inouye et al., 1993; Albert et al., 1995; Ylikoski et al., 1999; Habib et al., 2007), feeling healthy (von Faber et al., 2001; Andrews et al., 2002; Liang et al., 2003; Habib et al., 2007), race, income, gender (Inouye et al., 1993), physical activity, peak pulmonary flow rate, self-efficacy (Berkman et al., 1993; Albert et al., 1995), physical functioning, lifestyle, affect, personality (Andrews et al., 2002), and social functioning (von Faber et al., 2001). Only education and feeling healthy have been consistently identified in several studies as predictors of successful aging, which, as mentioned, takes into account the preservation of several cognitive functions and even other types of functions, such as physical and social.

Therefore, to improve our understanding of a specific cognitive domain, the present study focuses only on memory, particularly episodic memory. This type of memory refers to the ability to remember unique personal past experiences; thus, the memories for one person are different from those of other people for the same event (Tulving, 1972). There is consensus that most people undergo a gradual decline of their episodic memory functioning with advancing age (for a meta-analysis, see Verhaeghen and Salthouse, 1997). However, alongside this predominant fact, there is evidence that some individuals preserve their episodic memory abilities in older age. Although these particular individuals represent a crucial source of information to understand the mechanisms that contribute to the maintenance of such a vital function, little attention has been given to investigating the characteristics of this group of individuals.

Episodic memory encompasses the sense of chronesthesia, personal sense of time (Tulving, 2002), and autonoetic consciousness (the awareness of existing in our subjective time) (Tulving, 1985). These properties provide the experience of identity and self-continuity that allow us to distinguish between remembering from thinking, imaging, perceiving or daydreaming (Tulving, 1985). Thus, episodic memory goes beyond the ability to remember our own experiences and is essential to maintain our uniqueness and the ability to communicate with each other and perform functional everyday living activities. The importance of episodic memory is evident when memory starts to decline because individuals lose control of their lives and their autonomy, experiences that are often associated with emotional feelings of annoyance and frustration due to the impossibility to compensate for this memory deficit with other cognitive functions.

Moreover, with advancing age, older adults do not perceive that their entire cognitive constellation is starting to decline. Conversely, these individuals mainly complain about memory troubles, particularly episodic memory loss because this type of memory is highly vulnerable to the aging process. Additionally, episodic memory impairment is also the first symptom in Alzheimer’s disease (Bäckman et al., 2001) and traumatic brain injury (Wammes et al., 2017).

Although episodic memory decline increases in older age, few studies have examined predictors of episodic memory exclusively. One of them (Sundermann et al., 2016) evaluated the effects of diet on episodic memory decay and found no relationship. Another study (Josefsson et al., 2012) evaluated maintainers and decliners of episodic memory over a 15-year period and found that being a woman, having a higher education level, engaging in physical activity and living with someone were predictors of episodic memory stability. Given that little is known about which factors, habits or everyday life styles may be relevant to episodic memory, examined the predictors that allow some individuals to preserve their episodic memory and those associated with people whose episodic memory strongly declines.

Within episodic memory, the ability to retrieve events from memory along with the context in which they originally took place, such as the spatial, temporal or emotional contexts, is highly vulnerable to the effects of aging (Spencer and Raz, 1995). In the present study, we employed a source memory paradigm that examined the ability to retrieve spatial information because this kind of context pertains to all episodic experiences. The examination of the nature and amount of contextual information that is encoded along with item information are important variables in determining patterns of performance of episodic memory.

As mentioned, we focused on both ends of source memory variability in old age: individuals whose source memory failed and those whose source memory succeeded. We defined these extreme groups according to participants’ accuracy in retrieving spatial contextual information. Individuals who performed in the lower 20th and upper 80th percentiles were classified as belonging to the episodic memory failed and succeeded groups, respectively. The age range of the participants was 61–80 years because according to the United Nations (2015), the cutoff to refer to older persons is 60 years or more, and those aged above 80 years are further classified as oldest-old because these individuals are considered at higher risk of health conditions.

To search exclusively for predictors of successful and failed source memory, we used logistic regression analyses. A set of 120 potential predictors were investigated that included demographic characteristics, illnesses, biological, physical, and physiological measures, nutrient, medication and drug consumption, and physical, mental, social, and cultural activities, among others. We expected that biological variables, followed by consumption habits, and then by environmental variables would be, in this order, the strongest predictors of both episodic memory success and episodic memory failure in older age.

## Materials and Methods

### Participants

The study included 519 healthy adults between the ages of 61 and 80 (265 women and 254 men); their average age was 70.2 ± 5.4 years (mean ± SD), and they had 13.3 ± 4.5 years of formal education. This subsample was drawn from a larger sample based on the participants’ age (Cansino et al., 2018a). Participants were recruited in Mexico City through advertisements, appeals to community groups, flyers, and word of mouth. The inclusion criteria included a minimum of 8 years of education, normal or corrected-to-normal vision, no addiction to drugs or alcohol, no consumption of medications that act on the nervous system in the last 6 months, and absence of head trauma and neurological or psychiatric diseases. In addition, participants were required to obtain a score ≤ 20 on the Beck Depression Inventory (BDI) (Beck, 1987), a score ≥ 24 on the Mini-Mental State Exam (MMSE) (Folstein et al., 1975), and a score ≥ 26 on the vocabulary subtest of the Wechsler Adult Intelligence Scale-Revised (WAIS-R) (Wechsler, 1981) to assure that they were not suffering from depression, dementia, or intellectual difficulties. Participants’ characteristics and performance in the psychological tests are displayed in Supplementary Tables S1, S2. All participants provided informed consent and received a monetary reward for their participation. The study was approved by the Bioethics Committee of the School of Medicine at the National Autonomous University of Mexico and was conducted in accordance with the Declaration of Helsinki. A power analysis was conducted with the software G^∗^Power version 3.2.9.2 to determine the sample size required to conduct logistic regression analyses. The parameters used to calculate the sample size were one-tailed tests with an alpha level of 0.05, a power value of 0.90, and an expected *R*^2^ of 0.20, and the result suggested a total of 173 required participants.

### Instruments

#### Metamemory

Knowledge, affects and beliefs about the participants’ own memory were measured with the Metamemory in Adulthood (MIA) (Dixon et al., 1988) scale. The MIA assessed seven dimensions: use of memory strategies, knowledge of memory tasks, knowledge of one’s own memory capacities, perception of memory change, relationship between anxiety and memory performance, achievement on memory tasks, and locus of control in memory abilities. The MIA is composed of 108 questions and statements evaluating the degree of agreement with various sentences or the frequency of some behaviors through a 5-point Likert scale. The scale was translated into Spanish, and then the translated version was reviewed independently by 10 judges for linguistic and cultural validation, which consisted of assessing the equivalence of concepts in the questionnaire and adapting the concepts to the Mexican culture.

#### Stressful Life Events

Positive and negative stressful life events were assessed through the Social Readjustment Rating Scale (SRRS) (Holmes and Rahe, 1967). The scale comprised 43 stressful events, and for each item, participants were asked if they had experienced them in the last 12 months. The magnitude of readjustment required for each life event was originally estimated by a sample of 394 persons; however, because the values assigned to each event have been challenged, we scored the scale as the number of stressful events endorsed by each participant.

#### Nutrient Consumption

Dietary intake was measured with the Food Frequency Questionnaire (FFQ) (Hernández-Ávila et al., 1998), a semiquantitative instrument composed of 116 food items. For each item, the frequency of consumption over the previous year was measured using 10 frequency categories ranging from never, less than once a month, 1–3 times per month, once a week, 2–4 times per week, 5–6 times per week, once a day, 2–3 times per day, 4–5 times per day, and 6 times per day. The FFQ was developed in many steps following the method proposed by Willett et al. (1985). Initially, food items were identified from a random sample of families in Mexico City (Hernández et al., 1983) through 24-h recalls and home visits to weigh and measure the food items consumed. Then, dietitians and nutritionists identified possible food items that could be missing; afterward, the FFQ was built and piloted with a similar sample. Nutrient intake was estimated using the Evaluation System of Nutritional Habits and Nutrient Consumption (SNUT) software developed by the National Institute of Public Health (Hernández-Ávila et al., 2000). The nutrient content for each food item was estimated per average unit (serving size) from the US Department of Agriculture ((1963–1997), USDA) and complemented by a database from the National Institute of Nutrition (Chávez et al., 1996). The reproducibility of the FFQ was assessed for a period of 1 year, and its validity was estimated by comparing results from the FFQ with those obtained from four 4-day 24-h recalls during a period of 1 year (Hernández-Ávila et al., 1998) and with serum blood levels for some of the nutrients (Romieu et al., 1999). In addition, we asked participants how often they consumed canned food and processed food using the same frequency scale.

#### Lifestyle

An *ad hoc* Lifestyle Questionnaire was created for the current study to examine education, occupation, income, health status, medication intake, tobacco, drug and alcohol consumption, and cultural, social, mental and physical activities. Psychologists who were carefully trained before collaborating in the study applied the questionnaire as a semistructured interview. Income was classified into nine categories ranging from less than 1000 Mexican pesos per month to 30000 or more Mexican pesos per month. Retirement was measured as the time elapsed between retirement and the interview date. To examine health status, participants were requested to report the diseases that had been formally diagnosed by a physician during their lifetime. Diseases for the following systems were examined: nervous, respiratory, cardiovascular, immune, sensory, digestive, renal, reproductive, endocrine, musculoskeletal, hepatic portal, and sleep.

The consumption of pharmaceutical drugs from each of the following categories was assessed: antidepressants, neuroleptics, nootropics, hypnotics, anxiolytics, analgesics, amphetamines, and hormonal therapy. Participants were asked if they had taken the medicine for the typical purpose, or they were asked for the reasons the drugs are usually prescribed; the classification names listed above were not used in the interview. Participants were asked to provide the medication name, age of initial consumption and intake frequency and duration. Afterward, the medicines reported were categorized by pharmaceutical specialists.

Consumption of cannabis, hallucinogens, cocaine, tobacco, and alcohol was investigated by asking participants to communicate age of onset of consumption, frequency and duration of consumption and time since last consuming the drug. Additionally, for tobacco and alcohol, participants indicated the number of cigarettes and glasses of alcohol they usually had when they smoked and drank. Likewise, participants reported the frequency with which they drank different alcoholic beverages (beer, wine, liqueur and spirits). Frequency was classified into 10 categories (never, once a year, three times per year, six times per year, once per month, two or three times per month, one or two times per week, three or four times per week, almost every day and daily). The amount of alcohol consumption was calculated as total grams per week based on the following equivalences: 6 g of alcohol/200 mL beer, 9.6 g of alcohol/100 mL wine, 25 g of alcohol/50 mL liqueur, and 42 g of alcohol/50 mL spirit.

Participants reported the frequency and time spent engaging in physical activity (aerobic and anaerobic exercise), engaging in mental activity (watching television, listening to the radio, using the computer and reading), attending cultural events (film screenings, theater plays, exhibitions, concerts, conferences or courses), attending social events (parties or reunions) and engaging in hobbies. The same 10 frequency categories described above for drug intake were used for these variables. Additionally, participants were asked to report the type of exercise that they performed most frequently and the genres of television, radio and literature that they most often chose. Likewise, the kind of activity they most frequently performed on the computer was assessed.

### Stimuli

Color images representing natural and artificial common objects (50% from each category) were used in the source memory paradigm. Examples of the kind of images used have been published elsewhere (Cansino et al., 2002). The complete set comprised 122 images, 2 of which were presented at the beginning of the encoding and retrieval tasks and were not analyzed, and 12 images were employed in a practice session. From the remaining 108 images, 72 of them were randomly selected for each participant to be presented during the encoding phase (equal proportion of natural and artificial objects), and the entire set of 108 images was randomly presented at the retrieval phase. Images were presented on a white background screen, and each of them subtended vertical and horizontal visual angles ranging from 2.9° to 4.3°.

### Source Memory Paradigm

The paradigm was divided into two tasks, encoding and retrieval. During the first task, the screen was divided into quadrants where the images were randomly displayed with the same probability of appearing in each of the quadrants. From the axes dividing the screen, images were displayed at a distance ranging from 0.5° to 1.25°. The trial started with the presentation of an image for 1000 ms followed by a 3000 ms period when the quadrants remained on the screen. Participants were required to classify each image as a natural or artificial object by pressing one of two keys during the 3500-ms period after the onset of the stimulus. To make a response in the source memory paradigm, a response panel consisting of five keys was used, four keys were arranged in two columns of two rows each to be pressed by the index and middle finger, and the fifth key was located in the lower portion of the response panel to be pressed by the thumb. The four keys represented the quadrants of the screen. The two keys in the second row were used during the encoding task. In the following retrieval task, the images were presented in the center of the screen, which was not divided into quadrants. Images were presented for 1000 ms, and participants had the same time to respond as in the encoding task. Participants were asked to judge whether the image was new or old. If the image was old, they indicated the quadrant where the image was originally presented during the encoding task by pressing one of four keys that represented each quadrant of the screen. If the image was new, participants used their thumb to press the lower (fifth) key on the response panel. Participants were instructed to randomly select one of the four quadrants if they were confident that the image was old but were unable to remember its exact position.

### Procedure

Participants attended two sessions of approximately 2 h each. The experimental sessions were conducted by graduate psychologist collaborators who were carefully trained for several months. Collaborators were evaluated through a Gesell chamber while conducting interviews and applying psychological tests before being allowed to formally carry out the study. Likewise, collaborators were supervised and evaluated while they administered the memory tasks to ensure that the same procedure was applied to all participants. Prior to being invited to attend the first session, potential participants were checked through prescreening questions if they satisfied the inclusion criteria. The first session occurred in a silent room in which only the participant and the experimenter were present. Participants were further interviewed to confirm that they fulfilled the inclusion criteria. Afterward, the WAIS-R Vocabulary subtest, the MMSE and the BDI tests were applied, and the participants’ vision was examined. Participants who were eligible for the study were asked to provide their informed consent. Then, the Lifestyle Questionnaire was applied, followed by the FFQ and the SRRS, which were completed in a counterbalanced order. Afterward, the Annett Hand Preference Questionnaire (Annett, 1970) was administered. The MIA questionnaire was then explained and handed to the participants to be answered at home; they were also asked to record all foods they ate for 3 days using special formats (data not analyzed here). At the end of the session, participants’ weight and height were measured.

At the beginning of the second session, participants’ glucose, cholesterol and triglycerides were measured in a non-fasting state with the Accutrend Plus System (Roche Diagnostics, Rotkreuz, Switzerland). Then, participants performed a working memory task (data not shown) in addition to the source memory paradigm in a sound-dampened chamber. Participants initially performed a brief version of the encoding and retrieval tasks as training and learned how to use the response panel. The stimulus presentation and response recording were controlled by E-Prime software v1.0 (Psychological Software Tools, Pittsburgh, PA, United States). Participants performed the task seating in a high-back armchair 100 cm away from the monitor screen. The response panel, adapted for right- or left-handed participants, was located on a platform on the left or right armchair according to the participant’s handedness. Blood pressure and heart rate were measured with a digital upper arm sphygmomanometer (Hem-712C, Omron, Kyoto, Japan). Mean arterial pressure (MAP) was estimated as [(2 × diastolic blood pressure) + systolic blood pressure]/3. Skin conductance responses were recorded by placing an electrode in the annular and medium fingers of the non-dominant hand (data not presented here).

### Statistical Analysis

We measured a total of 120 variables that, according to previous empirical findings, have been associated with or had an effect on episodic memory, memory in general or cognition. None of the variables had missing values, and those with skewness exceeding ± 3 were natural log-transformed. All variables were examined for linearity and homoscedasticity. Note that data were not developed by any reduction technique, so they represent precise evidence of the domains of interest and can be directly compared with predictors from other studies. The 20th and 80th percentiles in source memory accuracy were used to define those individuals who experienced episodic memory failure and those who maintained episodic memory success, respectively; each group included 104 individuals from the entire sample. Differences between the episodic memory success and non-success groups on the potential dichotomous predictor variables were analyzed using Pearson’s χ^2,^ and continuous predictor variables were analyzed using independent sample *t*-tests. For variables with unequal variance between groups, degrees of freedom were corrected with Satterthwaite’s procedure. The same analyses were conducted to analyze differences between the episodic memory failure and non-failure groups on all potential predictors. Only those variables that significantly differed between the success and non-success groups and between the failure and non-failure groups were considered potential predictors and were further analyzed through logistic regression models.

Predictors of episodic memory success were identified by means of logistic regression analyses. The dependent variable in the first logistic regression analyses was individuals with source memory success and individuals with non-success. Source memory accuracy was estimated as the percent of recognition hits accompanied by a correct source response. Images that were incorrectly classified during the encoding task were excluded from the analyses. For the initial model building, the independent variables were those predictors that significantly differed between the source memory success and non-success groups in the previous analyses. The model was adjusted for age because it significantly differs between groups. Sex [χ^2^(1, *N* = 519) = 0.04, *p* = 0.84] and years of education [*t*(517) = −1.11, *p* = 0.27] were not adjusted given that they did not differ between the episodic memory success group (54 women and 50 men, 13.7 ± 4.2 years of education) and the rest of the sample (non-success group) (211 women and 204 men, 13.2 ± 4.5 years of education).

Then, a backward elimination procedure was applied; independent variables were deleted, one at a time, if they did not contribute to the regression equation (*p* > 0.05). Therefore, the final regression model included only significant predictors.

The same procedure was used to build the second logistic regression model to identify predictors of episodic memory failure. With the variation that the dependent variable was individuals whose source memory failed and individuals whose source memory did not fail. The initial model included those variables that significantly differed between failure and non-failure groups. As in the first model, only age was adjusted because sex [χ^2^(1, *N* = 519) = 1.25, *p* = 0.26] and years of education [*t*(517) = −0.09, *p* = 0.93] did not differ between the episodic memory failure group (48 women and 56 men, 13.3 ± 4.5 years of education) and the rest of the sample (non-failure group) (217 women and 198 men, 13.3 ± 4.5 years of education).

The sensitivity and specificity for each final model were examined using receiver operating characteristic (ROC) analyses and area under the curve (AUC). Finally, predictive probabilities with 95% confidence intervals were computed for all significant predictors from both logistic regression models. All analyses were conducted using Stata v. 13 (College Station, TX, United States).

## Results

**FIGURE 1:**
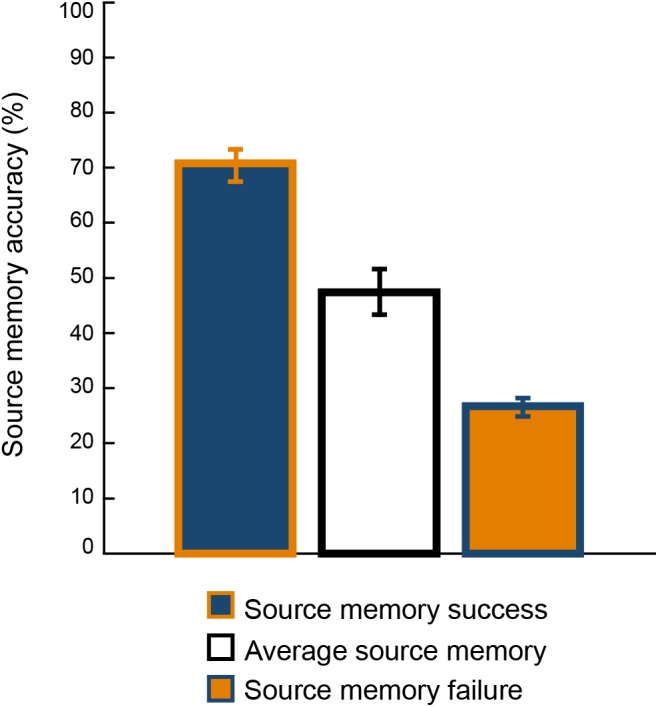
Mean performance in the source memory paradigm for the source memory success group (above the 80th percentile), for the source memory failure group (below the 20th percentile), and for individuals who performed between the extreme groups. Error bars represent the 95% confidence intervals for the mean.

The performance in the source memory paradigm for the episodic memory success group (above percentile 80th) and for the episodic memory failure group (below percentile 20th) is displayed in Figure 1; in addition, the mean source memory accuracy for individuals who performed between the extreme groups is depicted.

The results of the *t*-tests and χ^2^ analyses performed to identify potential predictors of source memory success on the initial set of 120 variables are displayed in Supplementary Table S1. These analyses revealed that only eleven variables significantly differed between success and non-success groups: scores on the BDI and on the anxiety and achievement scales of the MIA; cardiovascular system diseases; heart rate; diastolic blood pressure; MAP; consumption of anxiolytics, hormonal therapy and liqueur; and computer use. These variables were included in the first logistic regression model, but after performing backward elimination, only the following variables significantly predicted source memory success: scores on the BDI and the achievement scale of the MIA; heart rate; diastolic blood pressure; and hormonal therapy. The characteristics of the significant predictors are displayed in Table 1, and the results of the logistic regression model for episodic memory success are shown in Table 2.

**Table 1:** Characteristics of the significant predictors from each logistic model: At the top, predictors of source memory success; below, significant predictors of source memory failure.

	Non-success (*n* = 415) *M*(*SD*)	Success (*n* = 104) *M*(*SD*)	*t*-test *df* = 517	*P*
Age (years)	70.50 (5.42)	68.94 (5.25)	2.64	0.009^∗∗^
Beck Depression Inventory	7.43 (5.14)	5.96 (4.56)	2.67	0.008^∗∗^
MIA: Achievement	60.09 (6.64)	58.41 (6.73)	2.30	0.022^∗^
Heart rate (bpm)	71.61 (11.48)	69.03 (10.66)	2.08	0.038^∗^
Diastolic blood pressure (mmHg)^b^	4.32 (0.14)	4.36 (0.13)	−2.57	0.010^∗∗^
Hormonal therapy^ab^	0.39 (1.12)	0.94 (1.63)	−3.22	0.002^∗∗^

	**Non-Failure (*n* = 415) *M*(*SD*)**	**Failure (*n* = 104) *M*(*SD*)**	***t*-test *df* = 517**	***P***

Age (years)	69.89 (5.37)	71.37 (5.46)	−2.51	0.012^∗^
Retirement (years)^b^	1.21 (1.21)	1.57 (1.25)	−2.70	0.007^∗∗^
MIA: Strategy	57.91 (12.02)	54.86 (12.24)	2.31	0.021^∗^
MIA: Anxiety	43.24 (10.01)	45.63 (10.24)	−2.18	0.030^∗^
Glucose (g)^b^	3.01 (0.47)	3.11 (0.53)	−2.01	0.045^∗^
Beer (fr)^b^	0.25 (0.61)	0.12 (0.46)	2.42	0.017^∗^
Hobbies^ab^	1.14 (1.17)	0.86 (0.98)	2.58	0.011^∗^

*Age was controlled in both models. ^a^Total intake or time = frequency × duration. ^b^Log transformed variable. MIA, Metamemory in Adulthood Scale.*

**Table 2:** Logistic regression analysis results for source memory success.

Variable	Odds ratio	95% CI	*P*
Age (years)	0.957	0.917 – 0.999	0.044
Beck Depression Inventory	0.950	0.905 – 0.997	0.039
MIA: Achievement	0.964	0.932 – 0.997	0.032
Heart rate (bpm)	0.972	0.952 – 0.992	0.007
Diastolic blood pressure (mmHg)^b^	22.285	3.824 – 129.859	0.001
Hormonal therapy^ab^	1.414	1.207 – 1.656	<0.001

*^a^Total intake or time = frequency × duration. ^b^Log transformed variable. MIA, Metamemory in Adulthood Scale.*

The initial *t*-tests and χ^2^ analyses performed to identify potential predictors of source memory failure revealed that only eight out of 120 variables significantly differed between the source memory failure and non-failure groups (Supplementary Table S2). These variables were retirement; scores on the strategy and anxiety scales of the MIA; intermittent insomnia; consumption of carbohydrates, glucose and hormonal therapy; and hobbies. However, in the final logistic model, only six variables significantly predicted source memory failure: retirement; scores in the strategy and anxiety scales of the MIA; glucose and beer intake; and hobbies. Characteristics of these predictors are shown in Table 2, and the results from the final logistic regression model for source memory failure are depicted in Table 3.

**Table 3:** Logistic regression analysis results for source memory failure.

Variable	Odds ratio	95% CI	*P*
Age (years)	1.040	0.996 – 1.086	0.076
Retirement (years)^b^	1.261	1.042 – 1.525	0.017
MIA: Strategy	0.970	0.952 – 0.989	0.002
MIA: Anxiety	1.035	1.011 – 1.060	0.004
Glucose (g)^b^	1.857	1.154 – 2.989	0.011
Beer (fr)^b^	0.568	0.347 – 0.929	0.024
Hobbies^ab^	0.764	0.619 – 0.943	0.012

*^a^Total intake or time = frequency × duration. ^b^Log transformed variable. MIA, Metamemory in Adulthood Scale; fr, frequency.*

**FIGURE 2:**
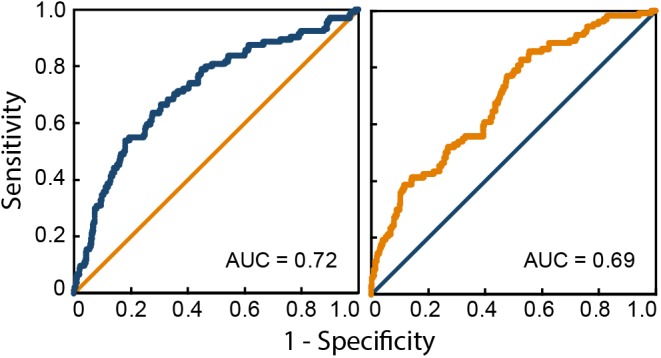
Receiver operating characteristics (ROC) curves for the source memory success model **(left)** and the source memory failure model **(right)**. AUC, area under the curve.

**FIGURE 3:**
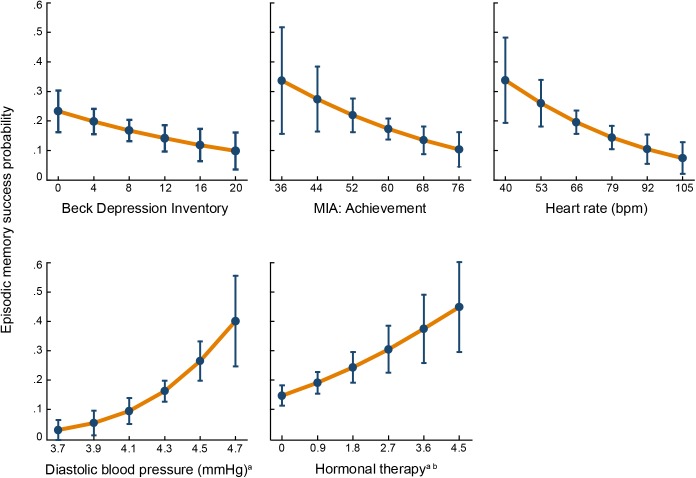
Predictive probabilities of source memory success for each significant predictor. Error bars represent 95% confidence intervals. MIA, Metamemory in Adulthood Scale. ^a^Log-transformed variable. ^b^Total intake = frequency × duration.

**FIGURE 4:**
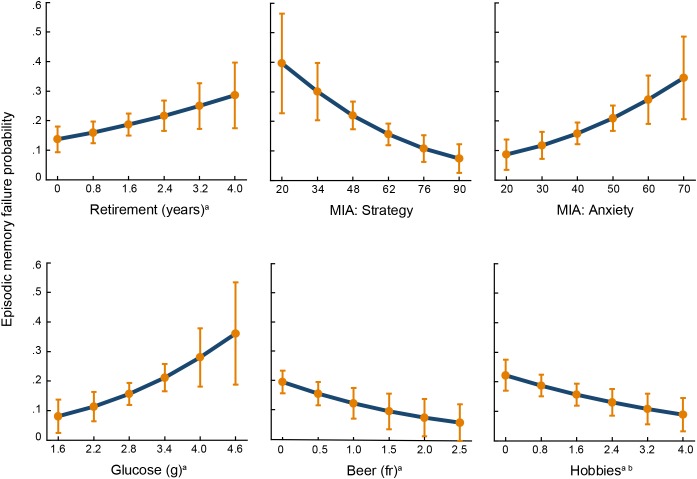
Predictive probabilities of source memory failure for each significant predictor. Error bars represent 95% confidence intervals. MIA, Metamemory in Adulthood Scale; fr, frequency. ^a^Log-transformed variable. ^b^Total time = frequency × duration.

Figure 2 shows the ROC curves for both logistic regression models. The AUC for the source memory success model was 0.72, *SE* = 0.03, 95% confidence interval (CI) = 0.66 – 0.77, and for the source memory failure model, it was 0.69, *SE* = 0.03, 95% CI = 0.63 – 0.75. Predictive probabilities for each significant predictor of source memory success are displayed in Figure 3, and those for significant predictors of source memory failure are depicted in Figure 4.

## Discussion

Individuals belonging to the episodic memory success group had mean source memory accuracy above 70%, performance that is comparable to that of persons less than 41 years old when completing the same source memory paradigm (Cansino et al., 2013). The mean source memory accuracy in the episodic memory failed group was only slightly above performance at chance level, which is 25% in a four-choice task. This group performed approximately 20% below individuals between 71 and 80 years old when executing the same task (Cansino et al., 2013). These results indicate that the 20th and 80th percentiles designated to identify both extreme groups discriminated appropriately.

The retainment of successful source memory in older age was predicted by the experience of fewer depression symptoms, less interest in performing efficiently in memory tasks, lower heart rate, higher diastolic blood pressure and higher use of hormonal therapy. Conversely, source memory failure in older age was predicted by having more years in retirement, employing fewer memory strategies, experiencing more anxiety when performing memory tasks, consuming more glucose in the diet, consuming less beer and spending less time on hobbies. The ROC curves demonstrated that both models are not random models (Figure 2). This was further supported by the lower value of the AUC’s 95% confidence intervals, which were higher than the 0.5 baseline. Below, we discuss the factors relevant to each of these logistic models.

### Predictors of Source Memory Success

As expected, one biological variable, diastolic blood pressure, was the strongest predictor of episodic memory success. According to worldwide trends (NCD Risk Factor Collaboration, 2017, [non-communicable diseases]), mean diastolic pressure was within normal ranges in individuals whose source memory was maintained (77.8, *SD* = 10.1) and in the rest of the participants (74.9, *SD* = 10.6). Moreover, systolic blood pressure was equivalent in both groups (mean = 127.5, *SD* = 17.9), which also corresponds to normal ranges since systolic blood pressure slightly increases with age (Drizd et al., 1986). This outcome indicates that diastolic blood pressure itself acts as a positive predictor of source memory success. As is well known, arterial pressure during diastole occurs between heart beats, when the heart receives blood and consequently oxygen, and at the same time, large vessels recoil and smoothly deliver blood to the periphery; hence, diastole is characterized by low pressure and low blood flow. In sum, diastolic pressure depends on the recoil of large vessels, the adequacy of the aortic valve and the resistance of peripheral vessels (Nair, 2016).

Although some studies have reported that high diastolic blood pressure is associated with cognitive impairment (e.g., Tsivgoulis et al., 2009) or with a higher risk of dementia (e.g., Skoog et al., 1996), this outcome has been observed mostly in individuals who have blood pressure at pathological levels (for a review see Reitz and Luchsinger, 2007). The inverse relationship has also been detected, and results from longitudinal studies (Qiu et al., 2003; Verghese et al., 2003) revealed that low diastolic blood pressure was associated with a higher risk of dementia. In the present study, higher diastolic blood pressure acted as a positive predictor of source memory success because it assured that cerebral blood flow was supplied even in resting heart state, a necessary condition for cognitive functioning. The logistic model indicated that there was a 22% increase in the odds of having successful source memory for a one-unit increase in diastolic blood pressure on a logarithmic scale, which corresponds to an increase in odds of 1.04% for a one-unit (mmHg) increase in diastole pressure. At higher blood pressure levels, the probability of source memory success increases, as shown in Figure 3. Low heart rate was the other biological predictor of source memory success. During heart contraction, the activation of baroreceptors, mainly in the aortic arch and carotid sinus, sends signals to the brain that reduce the amplitude of neural responses in the cortex, which interfere with orientation, attention and perception (Graham and Clifton, 1966; Lacey, 1967). This explains why memory processes that involve encoding stimuli benefits from slow heart rates (Jennings and Hall, 1980).

The second strongest predictor of source memory success was estrogen consumption. Notably, approximately 61% of women from the source memory successful group were on hormonal therapy, which corresponds to only 32% of the entire successful group including men, demonstrated the impact of this predictor on episodic memory. The administration of estradiol to gonadectomized female rats increased the activity of cholinergic neurons in the basal forebrain (Luine, 1985), the main producer of acetylcholine for the hippocampus and cerebral cortex (Woolf, 1991). Numerous studies in non-human and humans have confirmed the effects of estradiol on memory (for a review see Gibbs, 2010) due to its influence on hippocampus functioning, the main brain structure responsible for recollection (Brown and Aggleton, 2001).

Two individuals’ characteristics reliably predicted source memory success in older age, the experience of fewer depression symptoms and being less concerned about achieving memory control. Meta-analytic procedures (Burt et al., 1995; Zakzanis et al., 1998) that included numerous studies have provided strong evidence that individuals suffering from depression symptoms were also affected by significant memory deficits, mainly on episodic memory (Zakzanis et al., 1998). These findings are based on patients suffering from major depressive disorder and other clinically depressed patients; however, in healthy older adults, depression symptoms are also associated with memory impairments. Moreover, in a 12-year longitudinal study, it was found that depressive symptoms precede memory decline (Zahodne et al., 2014). In the present study, we found that individuals who showed high source memory performance reported significantly fewer depressive symptoms than participants whose source memory performance was at an average level or below. The achievement scale of the MIA measures one’s own motivation to perform well in memory tasks. Individuals from the source memory successful group were less disturbed by the idea of performing accurately in memory tasks than the other participants. This attitude benefited this group probably because more resources are designated to operate their memory instead of being dedicated to worrying about their memory.

### Predictors of Source Memory Failure

Contrary to our expectation, none of the biological variables predicted source memory failure. However, one consumption habit was the strongest predictor of episodic memory failure: the intake of the monosaccharide glucose, which, when combined with fructose, constitutes sucrose, the disaccharide used as table sugar. The World Health Organization (2015) proposed reducing sugar intake to 5% of total energy intake for a 2,000-calorie diet, which corresponds to 25 g per day. In the current study, mean glucose consumption in the memory failure group was precisely this amount without considering fructose and other glucose sources as carbohydrates; therefore, it is possible that their glucose consumption was underestimated. Glucose is essential for brain functioning, and several studies have demonstrated that a dose of 25 g of glucose enhances episodic memory (e.g., Boyle et al., 2018). However, glucose benefits on memory have an inverted-U dose response curve in elderly adults; thus, low and high glucose consumption has been associated with lower cognitive performance (Parsons and Gold, 1992). In the present study, the odds of source memory failure increased by a factor of 1.8 as glucose intake increased one unit on a logarithmic scale (Figure 4); this is equivalent to the odds of source memory failure of 1.03 for each one g increase in glucose consumption.

The other consumption habit associated with source memory failure was the lower amount of beer drinking. Similar to wine, beer contains numerous polyphenols, which have high antioxidant and anti-inflammatory properties that reduce the risk of cardiovascular diseases (Chen and Blumberg, 2009), which are largely associated with cerebrovascular dysfunctions that interfere with cognitive functionality. The benefits of beer consumption on the cardiovascular system have also been attributed to its alcohol content. Moderate alcohol consumption increased high-density lipoprotein cholesterol and diminished the accumulation of platelets (Arranz et al., 2012). In an adult life span sample, it was observed that beer consumption was a positive mediator of the effects of age on verbal working memory (Cansino et al., 2018b). The present outcome goes beyond these previous findings because it not only suggests that beer consumption may be beneficial for memory but also provides evidence that its consumption may be needed to avoid source memory capability loss. Moreover, this finding, together with those of the study above, indicates that moderate beer consumption is relevant to protect the decay of more than one type of memory.

Two closely related environmental variables were significant predictors of source memory failure, the elapsed time since retirement and the time and frequency dedicated to performing hobbies. The longer the time after retirement and the less time spent in hobbies, the greater the probability of belonging to the source memory failure group. A previous study (Bonsang et al., 2012) also found that retirement negatively impacted episodic memory; however, not all studies found retirement effects on cognitive decline (for a review see Meng et al., 2017). The inconsistent results may be attributed to the fact that other related factors are not always considered. The finding that two highly related experiences, retirement and hobbies, were significant predictors of source memory failure suggested that being less engaged in mental challenging activities was the actual underlying influence of these variables on source memory failure.

Additionally, two metamemory dimensions predicted source memory failure. Metamemory is conceived as the knowledge we have about our memory functioning (Flavell, 1979). This knowledge influences how we control and monitor memory processes (Nelson and Narens, 1990). Although the assessment of the relationship between metamemory and objective memory has encountered mixed results (Castel et al., 2012), in the current study, older adults who used less intrinsic and extrinsic strategies to compensate for memory loss abilities belonged to the source memory failure group. Additionally, individuals who experience more anxiety when using their memory had a higher probability of inefficiently employing their memory. Both of these metamemory dimensions suggested that source memory failure is associated with the loss of memory control processes.

### General Remarks

One of the limitations of the present study is that some of our variables were assessed through participants’ reports that may be affected by inaccuracies or memory imprecisions that produce random measurement errors. Another limitation of the study is that some of the participants’ responses may have been modified due to social desirability; however, this problem should also be randomly distributed among all participants. Additionally, because we used a cross-sectional design, the direction of causality between variables cannot be drawn. Although most of the variables that remained in the two final logistic models occurred before memory was tested or were manifested for a certain period of time before the study took place, their causal influence is uncertain at this point. Longitudinal studies are needed to confirm these cross-sectional findings. Another limitation of the study is that some of the participants from the failure group may have been affected by mild cognitive impairment because the inclusion criteria were not sufficiently sensitive to detect this condition.

The models estimated identified potential predictors of both possible extreme memory outcomes during older age: preserving an efficient and successful control of source memory abilities or failing to do so. The group that attained successful source memory was characterized by having efficient cardiovascular signs that are fundamental to ensure brain functioning and by being more adapted to the exigencies that aging entails, manifested by being non-depressed and less concerned about memory loss. Women in particular also benefited from hormonal therapy, as was observed previously in an adult life span sample (Cansino et al., 2018a). Conversely, source memory failure was predicted by high glucose consumption and low intake of beverages rich in antioxidant and anti-inflammatory proprieties. Beer consumption was more relevant than wine consumption as a source of these health benefits because in Mexico, beer per capita consumption is 3.96 compared with wine, which is only 0.02 (World Health Organization, 2011). In addition, source memory failure was predicted in individuals who are less engaged in mental activities due to retirement, lack of hobbies and less implementation of strategies to compensate for memory failures. Likewise, the feeling of anxiety when memory functions take place can significantly disturb the recollection of the details of our past experiences. Note that these models predict extreme source memory outcomes in older age that occur in individuals who performed higher and lower than 80% of the entire sample.

In summary, we have examined in the same cohort of individuals almost all possible predictors that have been empirically identified as having an effect on cognition. Notably, only a few of these variables significantly predicted either source memory success or failure. This finding indicates that episodic memory is predicted by very precise factors and not by numerous variables, such as cognition, an outcome that was obtained by exclusively examining one cognitive domain. Source memory success was mainly predicted by two signs of heart functioning, which indicates that optimal brain function depends on cardiac output to supply steady cerebral blood flow. By contrast, individuals whose brains are not challenged in cognitive activities have a major possibility of losing the ability to recollect past experiences.

## Data Availability

The raw data supporting the conclusions of this manuscript will be made available by the authors, without undue reservation, to any qualified researcher.

## Ethics Statement

This study was carried out in accordance with the recommendations of the Declaration of Helsinki and the Bioethics Committee of the School of Medicine at the National Autonomous University of Mexico, with written informed consent from all subjects.

## Author Contributions

SC conceptualized and designed the study. FT-T, CE-M, LR-B, MP-L, AN-C, and MH-LdG performed the experiments. SC and SR-V analyzed the data. SC wrote the paper.

## Conflict of Interest Statement

The authors declare that the research was conducted in the absence of any commercial or financial relationships that could be construed as a potential conflict of interest.
