# Supplementary material for: Predictors of Source Memory Success and Failure in Older Adults
Source: Front Aging Neurosci. 2019 Feb 5;11:17. doi: 10.3389/fnagi.2019.00017 (PMC6371062; doi:10.3389/fnagi.2019.00017)
Supplement: Supplementary file 1 [file Data_Sheet_1.PDF]

Supplementary Material

Predictors of Source Memory Success and Failure in Old Adults

Selene Cansino*, Frine Torres-Trejo, Cinthya Estrada-Manilla, Liuba Ramírez-Barajas, Miguel Pérez-Loyda, Aidé Nava-Chaparro, Mariana Hernández-Ladrón-deGuevara, Silvia Ruiz-Velasco

*** Correspondence:** Corresponding Author: selene@unam.mx

Supplementary Tables

Table S1. Potential predictors of source memory success.

Table S2. Potential predictors of source memory failure.

Table S1. Characteristics of all potential predictors of source memory success.

Variables	All (n = 519) M(*SD*)	Non-Success (n = 415) M(*SD*)	Success (n = 104) M(*SD*)	*t*-test [a]/χ^2 [b] *df* = 517/1	*P*
Age (years)	70.19 (5.42)	70.50 (5.42)	68.94 (5.25)	2.64	.009**
Sex (women/men)[b]	265/254	211/204	54/50	0.04	.844
Education (years)	13.28 (4.48)	13.17 (4.54)	13.71 (4.20)	-1.11	.268
Income (ranks)	4.93 (2.02)	4.86 (2.03)	5.20 (1.98)	-1.54	.123
Retirement (years)[d]	1.28 (1.23)	1.28 (1.24)	1.26 (1.16)	0.19	.848
Vocabulary scale (WAIS-R)	12.80 (1.83)	12.73 (1.86)	13.10 (1.66)	-1.86	.064
MMSE	28.18 (1.45)	28.13 (1.47)	28.38 (1.31)	-1.56	.119
Beck Depression Inventory	7.14 (5.06)	7.43 (5.14)	5.96 (4.56)	2.67	.008**
SRRS[d]	1.84 (0.59)	1.82 (0.60)	1.89 (0.56)	-1.07	.283
MIA: Strategy	57.30 (12.12)	57.09 (12.11)	58.14 (12.17)	-0.79	.428
MIA: Task	59.08 (6.37)	59.00 (6.51)	59.41 (5.78)	-0.60	.552
MIA: Capacity	52.56 (9.81)	52.45 (9.88)	52.99 (9.56)	-0.50	.618
MIA: Change	44.69 (12.14)	44.27 (11.99)	46.34 (12.63)	-1.55	.122
MIA: Anxiety	43.72 (10.09)	44.20 (10.19)	41.81 (9.49)	2.17	.031*
MIA: Achievement	59.76 (6.68)	60.09 (6.64)	58.41 (6.73)	2.30	.022*
MIA: Locus	35.67 (5.44)	35.51 (5.44)	36.30 (5.44)	-1.32	.187
Illness (number)[d]	1.68 (0.54)	1.69 (0.55)	1.63 (0.51)	1.04	.300
Hypertension (no/yes)[b]	358/161	284/131	74/30	0.29	.592
Arrhythmia (no/yes)[b]	507/12	404/11	103/1	1.05	.305
Myocardial infarction (no/yes)[b]	504/15	401/14	103/1	1.72	.189
Hypotension (no/yes)[b]	517/2	413/2	104/0	0.50	.478
Hypercholesterolemia (no/yes)[b]	475/44	380/35	95/9	0.01	.943
Presbyopia (no/yes)[b]	269/250	220/195	49/55	1.16	.282
Cataracts (no/yes)[b]	478/41	383/32	95/9	0.10	.750
Hypoacusis (no/yes)[b]	494/25	393/22	101/3	1.06	.303
Diabetes mellitus (no/yes)[b]	457/62	364/51	93/11	0.23	.630
Initial insomnia (no/yes)[b]	486/33	391/24	95/9	1.15	.283
Terminal insomnia (no/yes)[b]	496/23	392/23	104/0	6.03	.014*
Intermittent insomnia (no/yes)[b]	483/36	383/32	100/4	1.92	.165
CSD (no/yes)[b]	244/275	186/229	58/46	4.00	.045*
Body mass index (k/m2)[d]	3.31 (0.13)	3.31 (0.13)	3.32 (0.14)	-1.03	.303
Glucose (mg/dL)[d]	4.61 (0.43)	4.60 (0.44)	4.67 (0.38)	-1.53	.128
Cholesterol (mg/dL)[d]	5.24 (0.18)	5.25 (0.18)	5.23 (0.17)	1.11	.267
Triglycerides (mg/dL)[d]	5.33 (0.53)	5.33 (0.54)	5.34 (0.53)	-0.05	.957
Heart rate (bpm)	71.09 (11.36)	71.61 (11.48)	69.03 (10.66)	2.08	.038*
Systolic blood pressure (mmHg)[d]	4.85 (0.14)	4.84 (0.14)	4.86 (0.13)	-0.91	.366
Diastolic blood pressure (mmHg)[d]	4.33 (0.14)	4.32 (0.14)	4.36 (0.13)	-2.57	.010**
Mean arterial pressure (mmHg)[d]	4.54 (0.13)	4.54 (0.13)	4.57 (0.12)	-2.17	.031*
Protein (g)[d]	3.63 (0.44)	3.63 (0.45)	3.63 (0.42)	0.02	.986
Energy (kcal)[d]	7.63 (0.34)	7.64 (0.34)	7.62 (0.32)	0.46	.643

Carbohydrates (g)[d]	5.62 (0.36)	5.63 (0.36)	5.60 (0.34)	0.66	.507
Sucrose (g)[d]	3.66 (0.48)	3.65 (0.48)	3.68 (0.46)	-0.62	.537
Fructose (g)[d]	3.33 (0.46)	3.33 (0.46)	3.34 (0.46)	-0.22	.824
Glucose (g)[d]	3.03 (0.48)	3.03 (0.48)	3.03 (0.49)	-0.17	.869
Calcium (mg)[d]	6.58 (0.49)	6.60 (0.49)	6.53 (0.48)	1.29	.197
Magnesium (mg)[d]	5.82 (0.34)	5.82 (0.35)	5.80 (0.32)	0.37	.711
Phosphorus (mg)[d]	7.06 (0.38)	7.07 (0.38)	7.04 (0.36)	0.80	.422
Potassium (mg)[d]	8.10 (0.37)	8.10 (0.37)	8.11 (0.35)	-0.13	.893
Sodium (mg)[d]	7.43 (0.41)	7.43 (0.41)	7.40 (0.38)	0.81	.418
Nitrate (g)[d]	4.22 (0.55)	4.22 (0.56)	4.23 (0.50)	-0.29	.773
Iron (mg)[d]	2.60 (0.32)	2.60 (0.32)	2.59 (0.30)	0.46	.643
Copper (mg)[d]	1.25 (0.40)	1.26 (0.40)	1.24 (0.37)	0.46	.648
Manganese (mg)[d]	2.78 (0.88)	2.76 (0.87)	2.85 (0.91)	-0.93	.352
Selenium (mcg)[d]	3.51 (0.54)	3.50 (0.55)	3.56 (0.51)	-0.96	.339
Zinc (mg)[d]	2.90 (0.46)	2.90 (0.46)	2.88 (0.45)	0.52	.603
Retinol (UI)[d]	7.73 (0.62)	7.74 (0.63)	7.65 (0.60)	1.33	.184
Carotenes (UI)[d]	8.94 (0.64)	8.93 (0.63)	8.99 (0.67)	-0.73	.468
Alpha-carotene (mcg)[d]	5.79 (1.16)	5.76 (1.16)	5.90 (1.15)	-1.14	.256
Beta-carotene (mcg)[d]	8.10 (0.65)	8.09 (0.64)	8.14 (0.70)	-0.73	.464
Beta-cryptoxanthin (mcg)[d]	6.28 (1.06)	6.29 (1.04)	6.23 (1.13)	0.54	.592
Lutein & xanthine (mcg)[d]	7.36 (0.72)	7.36 (0.74)	7.38 (0.65)	-0.32	.749
Lycopene (mcg)[d]	8.52 (0.87)	8.50 (0.88)	8.61 (0.82)	-1.12	.261
Thiamine (mg)[d]	0.94 (0.23)	0.94 (0.23)	0.93 (0.23)	0.38	.706
Riboflavin (mg)[d]	0.98 (0.26)	0.99 (0.26)	0.95 (0.26)	1.36	.175
Niacin (mg)[d]	2.97 (0.33)	2.97 (0.33)	2.96 (0.31)	0.09	.925
Pantothenic acid (mg)[d]	2.50 (0.57)	2.51 (0.57)	2.47 (0.59)	0.53	.597
Vitamin B6 glycosylated (mg)[d]	1.68 (0.76)	1.70 (0.75)	1.60 (0.79)	1.17	.241
Folates (mcg)[d]	6.63 (0.71)	6.63 (0.72)	6.61 (0.67)	0.26	.799
Vitamin B12 (mcg)[d]	1.84 (0.54)	1.85 (0.55)	1.81 (0.47)	0.67	.503
Vitamin C (mg)[d]	5.35 (0.59)	5.35 (0.58)	5.33 (0.60)	0.32	.747
Vitamin D (UI)[d]	5.24 (0.65)	5.24 (0.66)	5.23 (0.61)	0.11	.911
Alpha-tocopherol (mg)[d]	2.47 (0.36)	2.47 (0.36)	2.46 (0.36)	0.31	.759
Beta-tocopherol (mg)[d]	0.50 (0.19)	0.50 (0.19)	0.50 (0.19)	0.07	.942
Gamma-tocopherol (mg)[d]	2.41 (0.52)	2.41 (0.53)	2.45 (0.47)	-0.69	.491
Delta-tocopherol (mg)[d]	1.02 (0.38)	1.01 (0.38)	1.06 (0.37)	-1.21	.226
Vitamin K (mcg)[d]	4.38 (0.54)	4.37 (0.56)	4.39 (0.49)	-0.35	.726
Alcohol (g)[d]	0.40 (0.64)	0.41 (0.66)	0.37 (0.56)	0.66	.512
Caffeine (g)[d]	3.97 (1.84)	3.91 (1.86)	4.21 (1.77)	-1.49	.138
Cholesterol (g)[d]	5.34 (0.58)	5.34 (0.57)	5.31 (0.61)	0.44	.662
Butyric acid (g)[d]	0.28 (0.19)	0.29 (0.19)	0.26 (0.19)	1.26	.208
Caproic acid (g)[d]	0.19 (0.14)	0.19 (0.14)	0.17 (0.14)	1.28	.203
Caprylic acid (g)[d]	0.11 (0.08)	0.11 (0.08)	0.10 (0.09)	1.22	.225
Capric acid (g)[d]	0.26 (0.16)	0.26 (0.16)	0.24 (0.16)	0.90	.368
Lauric acid (g)[d]	0.31 (0.18)	0.31 (0.18)	0.30 (0.18)	0.40	.689
Myristic acid (g)[d]	0.90 (0.36)	0.91 (0.36)	0.87 (0.35)	0.81	.419

Palmitic acid (g)[d]	2.46 (0.40)	2.46 (0.40)	2.46 (0.37)	-0.10	.918
Stearic acid (g)[d]	1.70 (0.38)	1.69 (0.39)	1.72 (0.36)	-0.51	.612
Palmitoleic acid (g)[d]	0.84 (0.25)	0.84 (0.26)	0.84 (0.24)	0.05	.960
Oleic acid (g)[d]	2.81 (0.45)	2.81 (0.46)	2.84 (0.40)	-0.67	.500
Gadoleic acid (g)[d]	0.13 (0.07)	0.13 (0.07)	0.13 (0.07)	0.16	.876
Linoleic acid (g)[d]	2.10 (0.42)	2.09 (0.42)	2.13 (0.39)	-0.72	.474
Alpha-linoleic acid (g)[d]	0.83 (0.27)	0.83 (0.28)	0.84 (0.24)	-0.23	.815
Eicosatetraenoic acid (g)[d]	0.06 (0.05)	0.06 (0.04)	0.06 (0.06)	-0.41	.683
Eicosapentaenoic acid (g)[d]	0.06 (0.06)	0.06 (0.06)	0.06 (0.05)	-0.64	.524
Docosahexaenoic acid (g)[d]	0.16 (0.12)	0.16 (0.12)	0.17 (0.10)	-0.55	.579
Canned food (fr)	5.22 (2.05)	5.27 (2.05)	5.00 (2.02)	1.23	.221
Processed food (fr)	4.30 (2.88)	4.42 (2.89)	3.84 (2.81)	1.85	.065
Antidepressants[c d]	0.13 (0.53)	0.15 (0.57)	0.08 (0.37)	1.49	.138
Hypnotics[c d]	0.06 (0.33)	0.06 (0.35)	0.05 (0.27)	0.28	.782
Anxiolytics[c d]	0.12 (0.51)	0.14 (0.56)	0.06 (0.26)	2.12	.034*
Hormonal therapy[c d]	0.50 (1.26)	0.39 (1.12)	0.94 (1.63)	-3.22	.002**
Analgesics[c d]	0.25 (0.86)	0.25 (0.88)	0.23 (0.77)	0.28	.779
Amphetamines[c d]	0.02 (0.22)	0.02 (0.21)	0.03 (0.27)	-0.06	.955
Cannabis[c d]	0.00 (0.07)	0.00 (0.00)	0.02 (0.17)	-1.42	.159
Tobacco[c d]	2.35 (2.47)	2.38 (2.47)	2.23 (2.48)	0.54	.592
Cigarettes (number)[d]	1.00 (1.15)	1.00 (1.15)	0.98 (1.17)	0.17	.867
Alcohol[c d]	1.98 (1.63)	1.98 (1.65)	1.98 (1.59)	-0.03	.978
Alcohol (gr/week)[d]	2.25 (1.90)	2.23 (1.88)	2.31 (1.98)	-0.35	.724
Beer (fr)[d]	0.23 (0.58)	0.22 (0.58)	0.24 (0.61)	-0.37	.713
Wine (fr)[d]	0.22 (0.58)	0.23 (0.59)	0.20 (0.55)	0.36	.720
Liqueur (fr)[d]	0.04 (0.26)	0.05 (0.28)	0.01 (0.07)	2.81	.005**
Spirit (fr)[d]	0.78 (0.85)	0.76 (0.84)	0.84 (0.89)	-0.87	.384
Physical activity[c d]	0.20 (0.61)	0.20 (0.61)	0.20 (0.65)	0.02	.987
Television[c d]	2.58 (0.75)	2.55 (0.77)	2.69 (0.64)	-1.81	.072
Radio[c d]	2.34 (1.12)	2.33 (1.13)	2.41 (1.08)	-0.65	.517
Computer use (fr)	2.48 (3.62)	2.32 (3.56)	3.13 (3.78)	-2.05	.041*
Reading[c d]	1.72 (0.93)	1.70 (0.95)	1.77 (0.83)	-0.60	.549
Hobbies[c d]	1.09 (1.14)	1.06 (1.10)	1.21 (1.26)	-1.21	.226
Cultural activities (fr)	3.51 (2.33)	3.42 (2.36)	3.88 (2.21)	-1.78	.076
Social activities (fr)	4.31 (2.07)	4.25 (2.11)	4.53 (1.91)	-1.22	.225

Note. [a] Variables with unequal variance, *t*-tests were conducted with Satterthwaite's degrees of freedom
[b] Dichotomous variable, χ^2 analyses are reported
[c] Total intake or time = frequency x duration
[d] Log transformed variable
fr = frequency, WAIS-R = Wechsler Adult Intelligence Scale-Revised, MMSE = Mini Mental Stare Examination, SRRS = Social Readjustment Rating Scale, MIA = Metamemory in Adulthood Scale, CSD = Cardiovascular system diseases

Table S2. Characteristics of all potential predictors of source memory failure.

Variables	All (n = 519) M(*SD*)	Non-Failures (n = 415) M(*SD*)	Failures (n = 104) M(*SD*)	*t*-test [a]/χ^{2} [b] *df* = 517/1	*P*
Age (years)	70.19 (5.42)	69.89 (5.37)	71.37 (5.46)	-2.51	.012*
Sex (women/men)[b]	265/254	217/198	48/56	1.25	.263
Education (years)	13.28 (4.48)	13.27 (4.47)	13.31 (4.53)	-0.09	.929
Income (ranks)	4.93 (2.02)	4.92 (2.02)	4.96 (2.01)	-0.19	.853
Retirement (years)[d]	1.28 (1.23)	1.21 (1.21)	1.57 (1.25)	-2.70	.007**
Vocabulary scale (WAIS-R)	12.80 (1.83)	12.88 (1.82)	12.49 (1.82)	1.94	.053
MMSE	28.18 (1.45)	28.23 (1.45)	27.97 (1.40)	1.63	.104
Beck Depression Inventory	7.14 (5.06)	7.17 (5.04)	7.00 (5.17)	0.31	.755
SRRS[d]	1.84 (0.59)	1.84 (0.59)	1.84 (0.63)	-0.03	.975
MIA: Strategy	57.30 (12.12)	57.91 (12.02)	54.86 (12.24)	2.31	.021*
MIA: Task[a]	59.08 (6.37)	59.36 (6.10)	57.95 (7.27)	1.83	.070
MIA: Capacity	52.56 (9.81)	52.67 (9.89)	52.14 (9.54)	0.48	.629
MIA: Change	44.69 (12.14)	44.76 (12.16)	44.38 (12.14)	0.28	.776
MIA: Anxiety	43.72 (10.09)	43.24 (10.01)	45.63 (10.24)	-2.18	.030*
MIA: Achievement	59.76 (6.68)	59.74 (6.73)	59.82 (6.51)	-0.10	.918
MIA: Locus	35.67 (5.44)	35.61 (5.54)	35.90 (5.03)	-0.49	.622
Illness (number)[d]	1.68 (0.54)	1.69 (0.54)	1.62 (0.55)	1.17	.243
Hypertension (no/yes)[b]	358/161	284/131	74/30	0.29	.592
Arrhythmia (no/yes)[b]	507/12	406/9	101/3	0.19	.664
Myocardial infarction (no/yes)[b]	504/15	403/12	101/3	0.00	.997
Hypotension (no/yes)[b]	517/2	413/2	104/0	0.50	.478
Hypercholesterolemia (no/yes)[b]	475/44	381/34	94/10	0.22	.641
Presbyopia (no/yes)[b]	269/250	213/202	56/48	0.21	.645
Cataracts (no/yes)[b]	478/41	382/33	96/8	0.01	.930
Hypoacusis (no/yes)[b]	494/25	393/22	101/3	1.06	.303
Diabetes mellitus (no/yes)[b]	457/62	369/46	88/16	1.46	.227
Initial insomnia (no/yes)[b]	486/33	386/29	100/4	1.38	.240
Terminal insomnia (no/yes)[b]	496/23	396/19	100/4	0.11	.746
Intermittent insomnia (no/yes)[b]	483/36	391/24	92/12	4.27	.039*
CSD (no/yes)[b]	244/275	194/221	50/54	0.06	.808
Body mass index (k/m2)[d]	3.31 (0.13)	3.31 (0.13)	3.31 (0.13)	0.35	.728
Glucose (mg/dL)[d]	4.61 (0.43)	4.60 (0.44)	4.64 (0.38)	-0.77	.439
Cholesterol (mg/dL)[d]	5.24 (0.18)	5.24 (0.18)	5.25 (0.18)	-0.45	.655
Triglycerides (mg/dL)[d]	5.33 (0.53)	5.32 (0.54)	5.38 (0.50)	-1.07	.287
Heart rate (bpm)	71.09 (11.36)	71.14 (11.61)	70.91 (10.32)	0.18	.858
Systolic blood pressure (mmHg)[d]	4.85 (0.14)	4.85 (0.14)	4.85 (0.14)	-0.41	.680
Diastolic blood pressure (mmHg)[d]	4.33 (0.14)	4.33 (0.14)	4.31 (0.14)	1.31	.191
Mean arterial pressure (mmHg)[d]	4.54 (0.13)	4.54 (0.13)	4.54 (0.13)	0.32	.748
Protein (g)[d]	3.63 (0.44)	3.63 (0.45)	3.64 (0.44)	-0.30	.766
Energy (kcal)[d]	7.63 (0.34)	7.62 (0.33)	7.67 (0.36)	-1.38	.168

Carbohydrates (g)[d]	5.62 (0.36)	5.61 (0.35)	5.69 (0.38)	-2.20	.028*
Sucrose (g)[d]	3.66 (0.48)	3.64 (0.47)	3.70 (0.50)	-1.15	.252
Fructose (g)[d]	3.33 (0.46)	3.31 (0.45)	3.40 (0.47)	-1.73	.083
Glucose (g)[d]	3.03 (0.48)	3.01 (0.47)	3.11 (0.53)	-2.01	.045*
Calcium (mg)[d]	6.58 (0.49)	6.57 (0.48)	6.62 (0.50)	-0.81	.418
Magnesium (mg)[d]	5.82 (0.34)	5.81 (0.34)	5.84 (0.37)	-0.93	.352
Phosphorus (mg)[d]	7.06 (0.38)	7.06 (0.37)	7.09 (0.39)	-0.87	.384
Potassium (mg)[d]	8.10 (0.37)	8.10 (0.37)	8.12 (0.38)	-0.59	.553
Sodium (mg)[d]	7.43 (0.41)	7.43 (0.40)	7.43 (0.45)	-0.03	.972
Nitrate (g)[d]	4.22 (0.55)	4.23 (0.56)	4.20 (0.52)	0.46	.648
Iron (mg)[d]	2.60 (0.32)	2.59 (0.31)	2.63 (0.34)	-1.10	.273
Copper (mg)[d]	1.25 (0.40)	1.25 (0.40)	1.26 (0.38)	-0.19	.852
Manganese (mg)[d]	2.78 (0.88)	2.77 (0.89)	2.78 (0.86)	-0.06	.951
Selenium (mcg)[d]	3.51 (0.54)	3.51 (0.55)	3.52 (0.51)	-0.09	.925
Zinc (mg)[d]	2.90 (0.46)	2.90 (0.46)	2.87 (0.44)	0.64	.522
Retinol (UI)[d]	7.73 (0.62)	7.73 (0.62)	7.72 (0.63)	0.08	.939
Carotenes (UI)[d]	8.94 (0.64)	8.95 (0.64)	8.94 (0.63)	0.08	.938
Alpha-carotene (mcg)[d]	5.79 (1.16)	5.79 (1.16)	5.78 (1.17)	0.09	.927
Beta-carotene (mcg)[d]	8.10 (0.65)	8.10 (0.66)	8.09 (0.62)	0.16	.871
Beta-cryptoxanthin (mcg)[a d]	6.28 (1.06)	6.26 (1.09)	6.35 (0.91)	-0.87	.384
Lutein & xanthine (mcg)[d]	7.36 (0.72)	7.37 (0.72)	7.35 (0.72)	0.20	.843
Lycopene (mcg)[a d]	8.52 (0.87)	8.55 (0.80)	8.42 (1.12)	1.14	.257
Thiamine (mg)[a d]	0.94 (0.23)	0.93 (0.23)	0.96 (0.26)	-1.03	.307
Riboflavin (mg)[d]	0.98 (0.26)	0.98 (0.26)	1.00 (0.27)	-0.67	.506
Niacin (mg)[d]	2.97 (0.33)	2.96 (0.32)	2.97 (0.35)	-0.21	.835
Pantothenic acid (mg)[d]	2.50 (0.57)	2.51 (0.58)	2.46 (0.52)	0.72	.475
Vitamin B6 glycosylated (mg)[d]	1.68 (0.76)	1.65 (0.76)	1.80 (0.72)	-1.80	.073
Folates (mcg)[d]	6.63 (0.71)	6.63 (0.71)	6.63 (0.70)	-0.01	.990
Vitamin B12 (mcg)[d]	1.84 (0.54)	1.83 (0.53)	1.88 (0.55)	-0.79	.429
Vitamin C (mg)[d]	5.35 (0.59)	5.34 (0.58)	5.37 (0.60)	-0.46	.648
Vitamin D (UI)[d]	5.24 (0.65)	5.22 (0.65)	5.30 (0.65)	-1.01	.311
Alpha-tocopherol (mg)[d]	2.47 (0.36)	2.47 (0.36)	2.44 (0.35)	0.72	.471
Beta-tocopherol (mg)[d]	0.50 (0.19)	0.49 (0.18)	0.51 (0.20)	-0.79	.428
Gamma-tocopherol (mg)[d]	2.41 (0.52)	2.41 (0.52)	2.43 (0.51)	-0.29	.775
Delta-tocopherol (mg)[d]	1.02 (0.38)	1.02 (0.37)	1.05 (0.40)	-0.68	.497
Vitamin K (mcg)[d]	4.38 (0.54)	4.38 (0.55)	4.36 (0.54)	0.39	.696
Alcohol (g)[d]	0.40 (0.64)	0.41 (0.64)	0.38 (0.63)	0.41	.683
Caffeine (g)[d]	3.97 (1.84)	4.02 (1.82)	3.81 (1.92)	1.01	.315
Cholesterol (g)[d]	5.34 (0.58)	5.33 (0.59)	5.36 (0.53)	-0.38	.703
Butyric acid (g)[d]	0.28 (0.19)	0.28 (0.19)	0.30 (0.18)	-0.70	.483
Caproic acid (g)[d]	0.19 (0.14)	0.18 (0.14)	0.19 (0.13)	-0.64	.525
Caprylic acid (g)[d]	0.11 (0.08)	0.11 (0.09)	0.12 (0.08)	-0.69	.493
Capric acid (g)[d]	0.26 (0.16)	0.26 (0.16)	0.26 (0.16)	-0.51	.610
Lauric acid (g)[d]	0.31 (0.18)	0.31 (0.18)	0.31 (0.18)	-0.06	.956
Myristic acid (g)[d]	0.90 (0.36)	0.89 (0.36)	0.92 (0.36)	-0.59	.559

Palmitic acid (g)[d]	2.46 (0.40)	2.46 (0.39)	2.45 (0.43)	0.23	.819
Stearic acid (g)[d]	1.70 (0.38)	1.70 (0.37)	1.68 (0.42)	0.59	.558
Palmitoleic acid (g)[d]	0.84 (0.25)	0.84 (0.25)	0.84 (0.26)	0.28	.777
Oleic acid (g)[d]	2.81 (0.45)	2.82 (0.44)	2.78 (0.48)	0.90	.370
Gadoleic acid (g)[d]	0.13 (0.07)	0.13 (0.07)	0.13 (0.07)	-0.08	.936
Linoleic acid (g)[d]	2.10 (0.42)	2.10 (0.41)	2.08 (0.45)	0.46	.648
Alpha-linoleic acid (g)[d]	0.83 (0.27)	0.84 (0.28)	0.82 (0.27)	0.68	.494
Eicosatetraenoic acid (g)[a d]	0.06 (0.05)	0.06 (0.05)	0.06 (0.03)	0.82	.412
Eicosapentaenoic acid (g)[d]	0.06 (0.06)	0.06 (0.06)	0.06 (0.06)	0.43	.664
Docosahexaenoic acid (g)[d]	0.16 (0.12)	0.16 (0.12)	0.16 (0.12)	0.19	.846
Canned food (fr)	5.22 (2.05)	5.18 (2.06)	5.39 (1.99)	-0.97	.331
Processed food (fr)	4.30 (2.88)	4.30 (2.87)	4.33 (2.96)	-0.10	.923
Antidepressants[a c d]	0.13 (0.53)	0.13 (0.51)	0.13 (0.63)	0.12	.907
Hypnotics[a c d]	0.06 (0.33)	0.06 (0.31)	0.06 (0.42)	-0.07	.941
Anxiolytics[a c d]	0.12 (0.51)	0.11 (0.49)	0.15 (0.58)	-0.65	.514
Hormonal therapy[a c d]	0.50 (1.26)	0.56 (1.31)	0.27 (1.01)	2.48	.014*
Analgesics[c d]	0.25 (0.86)	0.26 (0.88)	0.19 (0.76)	0.74	.460
Amphetamines[c d]	0.02 (0.22)	0.02 (0.23)	0.03 (0.21)	-0.10	.924
Cannabis[a c d]	0.00 (0.07)	0.01 (0.08)	0.00 (0.00)	1.43	.154
Tobacco[c d]	2.35 (2.47)	2.44 (2.46)	2.01 (2.48)	1.58	.115
Cigarettes (number)[d]	1.00 (1.15)	1.04 (1.16)	0.82 (1.09)	1.75	.080
Alcohol[c d]	1.98 (1.63)	2.00 (1.65)	1.89 (1.58)	0.65	.514
Alcohol (gr/week)[d]	2.25 (1.90)	2.24 (1.88)	2.28 (1.98)	-0.17	.866
Beer (fr)[a d]	0.23 (0.58)	0.25 (0.61)	0.12 (0.46)	2.42	.017*
Wine (fr)[d]	0.22 (0.58)	0.22 (0.59)	0.23 (0.57)	-0.08	.935
Liqueur (fr)[a d]	0.04 (0.26)	0.03 (0.23)	0.07 (0.34)	-1.00	.318
Spirit (fr)[d]	0.78 (0.85)	0.76 (0.85)	0.82 (0.86)	-0.61	.540
Physical activity[c d]	0.20 (0.61)	0.18 (0.59)	0.28 (0.68)	-1.47	.143
Television[a c d]	2.58 (0.75)	2.57 (0.73)	2.61 (0.83)	-0.52	.602
Radio[c d]	2.34 (1.12)	2.37 (1.13)	2.22 (1.08)	1.23	.218
Computer use (fr)	2.48 (3.62)	2.59 (3.67)	2.04 (3.40)	1.39	.166
Reading[c d]	1.72 (0.93)	1.69 (0.93)	1.83 (0.92)	-1.45	.149
Hobbies[a c d]	1.09 (1.14)	1.14 (1.17)	0.86 (0.98)	2.58	.011*
Cultural activities (fr)	3.51 (2.33)	3.51 (2.35)	3.52 (2.29)	-0.03	.974
Social activities (fr)	4.31 (2.07)	4.28 (2.06)	4.40 (2.13)	-0.53	.599

Note. [a] Variables with unequal variance, *t*-tests were conducted with Satterthwaite's degrees of freedom
[b] Dichotomous variable, χ^2 analyses are reported
[c] Total intake or time = frequency x duration
[d] Log transformed variable
fr = frequency, WAIS-R = Wechsler Adult Intelligence Scale-Revised, MMSE = Mini Mental Stare Examination, SRRS = Social Readjustment Rating Scale, MIA = Metamemory in Adulthood Scale, CSD = Cardiovascular system diseases
